# The Dynamic Thumb-in-Palm Pattern in Children with Spastic Cerebral Palsy and Its Effects on Upper Limb Function

**DOI:** 10.3390/children8010017

**Published:** 2020-12-31

**Authors:** Ja Young Choi, Dong-Wook Rha, Seon Ah Kim, Eun Sook Park

**Affiliations:** 1 Department of Rehabilitation Medicine, Severance Hospital, Research Institute of Rehabilitation Medicine, Yonsei University College of Medicine, Seoul 03722, Korea; jaychoi3399@gmail.com (J.Y.C.); medicus@yonsei.ac.kr (D.-W.R.); 2 Department of Rehabilitation Medicine, Chungnam National University College of Medicine, Daejeon 35015, Korea; 3 Department of Pediatric Occupational Therapy, Severance Rehabilitation Hospital, Seoul 03722, Korea; SENSORY@yuhs.ac

**Keywords:** hand deformity, thumb, cerebral palsy, upper limb, function

## Abstract

The thumb-in-palm (TIP) pattern is one of the most common upper limb deformities in cerebral palsy (CP). This study was designed to investigate the effect of the dynamic TIP pattern on upper limb function in children with spastic CP. This prospective observational study included a total of 106 children with CP with dynamic TIP. The House TIP classification while grasping small or large objects, Melbourne Assessment of Unilateral Upper Limb Function (MUUL), Shriners Hospital Upper Extremity Evaluation (SHUEE), Zancolli classification for wrist–finger flexor deformity, and degree of swan neck deformity were assessed. Type I was the most common and highest functioning House TIP classification type. However, there were no significant differences in upper arm function between types II, III, and IV. The three components of the SHUEE showed stronger association with MUUL than House TIP and Zancolli classifications. After multivariable analysis, functional use of the wrist–finger and the thumb played a more significant role than the dynamic alignment of the thumb. In conclusion, the House TIP classification is useful to describe the TIP pattern. The SHUEE thumb assessment is a useful tool for reflecting upper arm function. The upper arm function was related more with the associated wrist flexor deformity than dynamic TIP.

## 1. Introduction

Cerebral palsy (CP) is the most prevalent childhood disorder involving movement and postural control [1]. Spastic CP is the most common form. Children with spastic CP demonstrate spasticity of the upper limbs, which produces characteristic postures and deformities such as shoulder internal rotation, elbow flexion, forearm pronation, and thumb-in-palm (TIP) or finger flexion [2]. The TIP deformity is one of the most common upper limb deformities in CP. Approximately 40.6–53.4% of patients with spastic CP have a TIP deformity in at least one hand [3,4].

The House TIP classification is a well-known surgical classification for static TIP deformities [5]. There are four types of static TIP deformities under this classification that are defined as types I–IV. Previous studies have also classified types of “dynamic” TIP deformities according to the House TIP classification [3,4]. House TIP type IV pattern involves more muscle groups and appears to be more severe than House TIP type I [4]; however, the severity between House TIP types II, III, and IV is still undefined. Therefore, it is unknown whether House TIP classifications reflect the severity of dynamic TIP for upper limb function.

The Shriners Hospital Upper Extremity Evaluation (SHUEE) is a useful and validated tool for assessing upper limb function [6]. The SHUEE (thumb) assessment provides detailed information regarding the spontaneous use of the thumb and dynamic segment alignment of thumb on-demand use [6].

The thumb has a critical role in hand function, especially for grasping and pinching [7]. Therefore, spastic TIP deformity severely restricts upper limb function. However, the negative impact of TIP on upper limb function has never been extensively studied. Therefore, this study investigated the impact of dynamic TIP patterns on upper limb function and the usefulness of House TIP classifications and the SHUEE thumb assessment for dynamic TIP in children with spastic CP.

According to previous studies [3,4], dynamic wrist–finger flexor deformities, measured by the Zancolli classification system, are significantly associated with House TIP classification type, and swan neck deformity is the most common finger deformity. These deformities can also adversely affect function. Therefore, another aim was to investigate the influence of these upper arm deformities associated with dynamic TIP patterns on upper arm function in children with spastic CP.

## 2. Materials and Methods

### 2.1. Study Design

This study was a prospective, cross-sectional observational study. The guidelines of the STROBE statement (Strengthening the Reporting of Observational Studies in Epidemiology) were used for this report [8]. Ethical approval was granted by the Institutional Review Board (IRB) and Ethics Committee of Severance Hospital (#4-2012-0265). Informed consent for participation was obtained from the parents of all children in this study according to the rules of the IRB of our hospital. In addition, oral or written assent was also obtained from the children over 7 years old according to their understanding and cognitive abilities.

### 2.2. Participants

This study was conducted in a rehabilitation hospital affiliated with the university. Between September 2013 and February 2016, 472 children with spastic CP admitted to Severance Hospital for intensive therapy were consecutively screened for inclusion and exclusion criteria. The inclusion criteria of study subjects were: (1) a medical diagnosis of spastic CP confirmed by the hypertonia assessment tool; (2) a dynamic TIP deformity with adducted thumb in resting position or while grasping a small or large object and not static TIP contracture; (3) between 3 and 15 years of age; and (4) an ability to maintain a stable sitting position while reaching for an object. The exclusion criteria of study subjects were: (1) an inability to understand and follow commands; (2) previous orthopedic surgery on an upper extremity; and (3) chemodenervation therapy in the upper extremity within the last 6 months.

Following these criteria, 106 children with spastic CP were recruited for this study. The more severely affected hand was assessed in each subject. The mean age was 7.3 years (standard deviation ± 3.5 years). The GMFCS (Gross Motor Function Classification System) is widely used to assess the gross motor functions of children with CP, and the MACS (Manual Ability Classification System) was developed to evaluate typical manual performance in daily life [9,10]. Therefore, the general characteristics, distribution of GMFCS and MACS levels of the children study participants are presented in Table 1.

### 2.3. Measurements

Upper limb deformities and function were measured according to the following assessment tools. Each examination was also video recorded for subsequent scoring.

#### 2.3.1. Assessments for Upper Limb Deformities

##### Thumb-in-Palm Pattern Assessment

**Figure 1:**
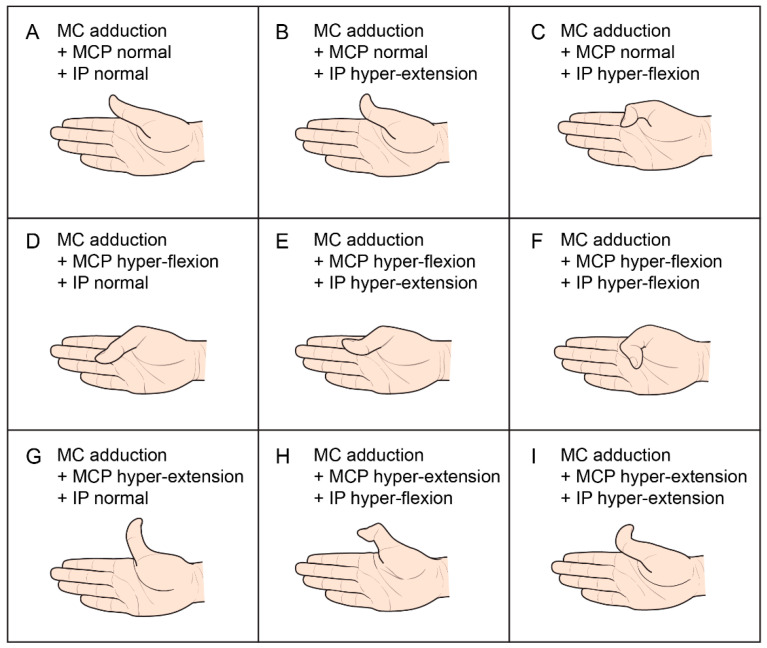
Thumb-in-palm deformities according to each joint level. MC, metacarpal; MCP, metacarpophalangeal; IP, interphalangeal joints; A = Ia, B = Ib, C = Ic, D = IIa, E = IIb, F = IV, G/H/I = III House thumb-in-palm classification.

The House TIP classification has four types: type I, metacarpal (MC) adduction; type II, MC adduction and metacarpophalangeal (MCP) joint flexion; type III, MC adduction and MCP joint hyperextension; and type IV, MC adduction and MCP and interphalangeal (IP) joint flexion [5]. The type I pattern is subdivided according to the condition of the IP joint: neutral (type Ia); hyperextension (type Ib); and hyperflexion (Ic). Type II House TIP classification can be further subdivided into two groups: IP joint neutral (type IIa) or hyperextension (type IIb). The type IV pattern can be differentiated from type II by the presence of IP joint flexion. The dynamic TIP deformities were assessed while the children were grasping a large object (hand-sized ball) or a small object (bean-sized pellet) in a sitting position. The dynamic TIP pattern while holding an object was assessed at each joint as follows: (1) MC joint; adduction or not; (2) MCP joint; normal, hyperflexion, or hyperextension; and (3) IP joint; normal, hyperflexion, or hyperextension (Figure 1). In addition, the presence of thumb MCP joint instability with hyperextensible laxity was also assessed.

##### Zancolli Classification

The Zancolli classification system is a useful system to classify wrist and finger flexor deformities [11]. In this study, we added level 0 to the Zancolli classification system in order to differentiate the normal pattern from the minimal wrist and finger flexion spastic pattern. Wrist and finger deformities were then defined: level 0, no flexion spasticity; level 1, minimal flexion spasticity; level 2a, moderate flexion spasticity with the ability to extend wrist; level 2b, moderate flexion spasticity with the inability to extend wrist; and level 3, severe flexion spasticity [3,12].

##### Swan Neck Deformity

The swan neck deformity was classified into three groups according to a previously used method: grade 1, no proximal IP hyperextension; grade II, proximal IP hyperextension (0° to 30°); and grade 3, >30° hyperextension with locking [13]. Zancolli classification system and swan neck deformity were assessed by a pediatric physiatrist (Choi JY).

#### 2.3.2. Assessments for Upper Limb Function

##### Melbourne Assessment of Unilateral Upper Limb Function

The MUUL (Melbourne Assessment of Unilateral Upper Limb Function) is a reliable and valid tool to measure the unilateral quality of upper limb movement based on activities such as reaching, grasping, releasing, and manipulation of the tool [14]. A total of 16 items are scored under four main categories: range of movement, target accuracy, fluency, and quality of movement [15]. In the present study, the total scores and the dexterity scores of the MUUL, including grasp, release, and manipulation tasks, were measured by an occupational therapist (Kim SA). In children younger than 5 years of age, the modified version of the MUUL was used. The modified version comprises minimal adjustments to the scoring of two test items, grasp and manipulation, considering maturational changes expected in children younger than 5 years of age [16].

##### Shriners Hospital Upper Extremity Evaluation

The SHUEE is the only known test that provides a detailed analysis of the dynamic alignment of the involved upper limb and its functional use [17]. It has been validated in children with CP between the ages of 3 and 18 years [6]. The assessment has three components: spontaneous functional analysis (SFA), dynamic positional analysis (DPA), and grasp and release analysis (GRA).

In this study, the SHUEE was assessed by an occupational therapist (Kim SA). The DPA (thumb and finger) and the thumb SFA were measured based on the performance of four selected tasks: retrieving money from a wallet, folding paper, tearing paper, and stringing beads. The maximum possible DPA (thumb) score is 12, DPA (finger) score is 12, thumb SFA score is 20, and GRA score is 6. Scores were assigned to each subscale and converted into percentages.

### 2.4. Statistical Analysis

Statistical analyses were performed using the Statistical Package for the Social Sciences for Windows (SPSS version 23, IBM SPSS Incorporated, Chicago, IL, USA). The Kruskal–Wallis test was performed to compare differences in MUUL score and SHUEE percentage among the House TIP groups. The post hoc Bonferroni correction was used for multiple comparisons between groups. Additionally, either Pearson’s correlation for parametric data or Spearman’s correlation rank analysis for nonparametric data was used to investigate associations among MUUL score, SHUEE percentage, TIP severity, and other functional hand classifications. The correlation coefficients were categorized as excellent (*r* ≥ 0.80), good (0.60 ≤ *r* < 0.80), fair (0.40 ≤ *r* < 0.60), or poor (*r* < 0.4). Additionally, univariate and multivariate linear regression models were used to identify factors significantly associated with MUUL score. Linear regression analysis was conducted separately between SHUEE and MUUL and between arm deformities (House TIP and Zancolli classification) and MUUL due to multicollinearity. A *p*-value less than 0.05 was considered significant in all statistical tests.

## 3. Results

### 3.1. Patterns of Dynamic Thumb-in-Palm Deformities

The TIP patterns for grasping large and small objects are presented in Table 2. Among the children that demonstrated TIP patterns while grasping a small object, 41 children (38.7%) had a normal pattern while grasping a large object. Type I of the House TIP classification was the most predominant TIP pattern. The IP joint hyperextension (type Ib) was most commonly noted in type I while the child was grasping a small object. In contrast, the normal IP joint (type Ia) pattern was most commonly noted while the child was grasping a large object. Among those with House TIP type II, IP joint hyperextension (type IIb) was the most common pattern. Type IV of the House TIP classification was observed in two children while grasping a large object and in six children while grasping a small object. Types I, II, and IV of the House TIP classification were more evident while the child was grasping a small object rather than a large object. In contrast, the number of cases demonstrating the type III pattern was higher while the child was grasping a large object rather than a small object (Table 2).

Thumb MCP joint instability was observed in 15 children (15.4%). All of the children (two children) with type III had MCP joint instability. In addition, seven children with type I (9.6%), four children with type II (16.0%), and two children with type IV (33.3%) had MCP joint instability.

### 3.2. Upper Limb Function According to the Pattern of Dynamic Thumb-in-Palm

Dynamic TIP became more evident while the child was grasping a small object rather than a large object. Therefore, hand function according to TIP pattern was analyzed based on the respective pattern while the child was grasping a small object.

In the type I House TIP classification, the dexterity and total MUUL scores and SHUEE DPA (thumb) were significantly different between types Ia, Ib, and Ic. The post hoc analysis revealed a significant difference in MUUL total scores between types Ib and Ic. However, there were no significant differences in MUUL, SHUEE DPA (thumb), SHUEE DPA (finger), or SHUEE GRA between types IIa and IIb of the House TIP classification.

The dexterity and total MUUL scores were significantly different between the four types of House thumb classifications. In addition, SHUEE DPA (thumb), SHUEE DPA (finger), SHUEE SFA, and SHUEE GRA were significantly different between the four types of TIP. Post hoc analysis revealed that dexterity, along with total MUUL scores and SHUEE scores of DPA (thumb), DPA (finger), SFA, and SHUEE GRA were significantly higher in type I than they were in types II and IV House TIP classifications. However, there were no significant differences in these parameters between types II, III, and IV House TIP classifications.

### 3.3. Relationships between Measurements

The MUUL scores showed a good relationship with Zancolli (*r* = 0.535 with dexterity, 0.544 with total, *p* < 0.01), a fair relationship with House TIP classification (*r* = 0.433 with dexterity, 0.441 with total, *p* < 0.01), but a poor relationship with swan neck deformity (*r* = 0.274 with dexterity, 0.253 with total, *p* < 0.01). In univariate linear regression analysis, House TIP (*R*^2^ = 0.221 with dexterity, 0.247 with total, *p* < 0.01), Zancolli classification (*R*^2^ = 0.350 with dexterity, 0.631 with total, *p* < 0.01), and swan neck deformities (*R*^2^ = 0.099 with dexterity, 0.091 with total, *p* < 0.05) were significantly related to MUUL scores. However, only the Zancolli classification continued to be significantly associated with MUUL scores after multivariate analysis (*R*^2^ = 0.390 with dexterity MUUL, 0.441 with total MUUL, *p* < 0.01).

In addition, the MUUL scores showed good relationships with the SHUEE DPA (thumb) (*r* = 0.676 with dexterity, 0.696 with total), SFA (*r* = 0.709 with dexterity, 0.740 with total), and GRA (*r* = 0.608 with dexterity, 0.635 with total), and a fair relationship with the SHUEE DPA (finger) (*r* = 0.480 with dexterity, 0.470 with total). In univariate linear regression analysis, SHUEE DPA (thumb and finger), SFA, and GRA were significantly related to MUUL dexterity score, and SHUEE SFA and GRA were significantly associated with MUUL total scores after multivariate analysis (*R*^2^ = 0.549 with dexterity MUUL, 0.598 with total MUUL) (Table 3).

## 4. Discussion

### 4.1. Patterns of Dynamic TIP Classifications and Upper Arm Function

This study demonstrates that dynamic TIP is more evident when grasping a small object than a large object, except in the type III pattern of the House TIP classification. Instability at the MCP joint is characteristic of the type III House thumb classification [13]. In our study, MCP joint instability was observed in all children with type III classification but was also observed in other types of House TIP classification. The effect of MCP joint instability on upper limb function was not examined in our study due to the small sample size. However, MCP joint instability may benefit from different therapeutic strategies than other dynamic TIP deformities. Therefore, we suggest that combined MCP joint instability should be carefully assessed in all types of TIP patterns.

In this study, dynamic TIP was classified into the four major types of House TIP classification. Types I or II of the House TIP classification were the predominant TIP pattern, which are in line with previous studies [3,4]. Types I to III can be further subdivided considering the condition of the IP joint. However, the significant differences in MUUL scores were only noted between types Ib and Ic. Hyperflexion of the thumb IP joint negatively affected upper limb function. However, IP joint hyperextension made it difficult to grasp laterally, in which the thumb contracts the lateral aspect of the index finger. The results suggest that the condition of the IP joint should be considered in type I for better therapeutic planning, such as selecting the target muscles for botulinum toxin injection.

House type I was the most highly functioning form of TIP classification, in which only the adductor pollicis muscle is involved in the deformity [4]. However, there were no significant differences in MUUL between types II, III, and IV. Although the difference may have been undetected given the small number of type III or IV cases, we believe the House TIP classification has limited ability to reflect functional severity. Further studies with larger numbers of children participants with types III and IV are needed to obtain conclusive evidence.

### 4.2. Associated Upper Arm Deformities

In children with spastic CP, TIP is commonly associated with other upper limb deformities [3,4]. In our study, two-thirds of the children had wrist and finger flexor spasticity (Zancolli classification ≥ 1). In contrast, swan neck deformity was observed in fewer than one-third of the children. According to a previous study, wrist flexion deformity is far more problematic than wrist pronation contracture or swan neck deformity [18]. The results of our study also demonstrate the more negative impact of the dynamic wrist–finger flexor deformity on upper arm function than dynamic TIP. These findings suggest that the presence of an associated dynamic wrist–finger flexor deformity should be assessed in therapeutic planning for dynamic TIP.

### 4.3. SHUEE Thumb Assessment

The SHUEE is a relevant tool for evaluation of spastic upper limb in CP that supports the indication of an intervention and measures its effects [14]. Significant improvement in SHUEE scores after surgical intervention for spastic upper arm in CP were reported in previous studies [6,19].

A systematic review considered the MUUL and SHUEE as useful for measuring the change in unimanual function as a result of spastic management [14]. As far as we know, there is only one report in terms of the validity of the SHUEE with other upper arm activity measures. In that study, a fair correlation was found between SFA and the self-care scaled score of the Pediatric Evaluation of Disability Inventory (PEDI) (*r* = 0.47) and a good inverse correlation between SFA and non-dominant total time of the Jebson–Taylor Test of Hand Function (*r* = −0.76) [6]. This was the first study to show the relationship between the SHUEE and MUUL.

The upper arm function was related more with the SHUEE DPA (thumb) than House TIP classification. These findings might be because the degree of MC abduction or extension while performing selected tasks was included in the scoring of DPA (thumb). In a previous report, TIP with clasp hand pattern was associated with the greatest functional disability among upper arm deformities [18]. In that study, TIP was defined as the inability to abduct the thumb past the level of the index finger due to thumb adductor contracture. These findings suggest that the degree of abnormal TIP pattern rather than the TIP pattern itself adversely affects upper arm function. The SHUEE DPA (thumb) is scored based only on the degree of the thumb MC adduction while performing four selected tasks [6], and thus it seemed to demonstrate a stronger association with upper arm function than House TIP classification. These findings are concurrent with the results of a previous study [20] in which static thumb alignment based on the House scale was not a good predictor of dynamic function after surgical intervention for thumb in palm deformity.

SHUEE GRA assesses the ability to perform grasp and release of digits with the wrist held in flexion, neutral, and extension positions [6], which presumably relates to the wrist–finger flexor tone. SHUEE SFA thumb reflects the spontaneous use of the thumb while performing tasks. The results of multivariable analysis showed that GRA and SFA of the thumb were the factors related to upper arm function but not to DPA thumb. These findings indicate that the functional use of wrist–finger and thumb play more important roles in upper arm function than the dynamic alignment of the thumb.

### 4.4. Study Limitation

The limitation of this study involves the unequal distributions of House TIP classification types. In this study, children who were not able to understand and follow the given commands were excluded. Therefore, there were too few cases of types III and IV to delineate the severity of TIP between types II and III and between types III and IV. Future studies with more type III and IV cases are needed to investigate the severity of TIP patterns regarding function.

## 5. Conclusions

The House TIP classification is useful for categorizing the abnormal patterns of TIP. The type I pattern of House TIP classification is the most highly functioning pattern among the four classification patterns. However, the severity of TIP patterns from type II to type IV regarding hand function is still under question. Although dynamic TIP adversely affects upper arm function, the negative impact of the dynamic wrist flexor deformity was greater than dynamic TIP. SHUEE thumb assessment is a useful tool to reflect upper arm function. In addition, the functional use of wrist–finger and thumb play more important roles in upper arm function than the dynamic alignment of the thumb. These findings suggest that a combined use of House TIP classification and SHUEE DPA (thumb) and the presence of associated dynamic wrist–finger flexor deformities are helpful for the therapeutic planning of dynamic TIP in children with spastic CP.

## Figures and Tables

**Table 1:** Baseline characteristics.

Characteristics	106 Children, 106 Hands
Sex, male: female	61: 45 (57.5: 42.5)
Evaluated hand, right: left	54: 52 (50.9: 49.1)
Assessment age (years)	7.3 ± 3.5 (3–15)
Type of cerebral palsy	
Unilateral	29 (27.4)
Bilateral	77 (72.6)
GMFCS, I: II: III: IV: V	31: 24: 36: 15: 0 (29.2: 22.6: 34.0: 14.2: 0)
MACS, I: II: III: IV: V	28: 48: 20:10: 0 (26.4: 45.3: 18.9: 9.4: 0)
Zancolli classification, 0: 1: 2A: 2B: 3	36: 54: 12: 4: 0 (34.0: 50.9: 11.3: 3.8: 0)
Swan neck classification, 1: 2: 3	78: 18: 10 (73.6: 17.0: 9.4)

Values are expressed as mean ± SD (range) or number of participants (percentage); GMFCS, Gross Motor Functional Classification System; MACS, Manual Ability Classification System.

**Table 2:** Thumb-in-palm pattern when grasping large or small objects.

MC	MCP	IP	House Thumb	Modified Classification	Number of Hands	
					Large Object	Small Object
Adduction	Normal	Normal	I	Ia	n = 33	n = 28
		Hyperextension	-	Ib	n = 9	n = 41
		Hyperflexion	-	Ic	n = 2	n = 4
	Hyperflexion	Normal	II	IIa	n = 4	n = 5
		Hyperextension	-	IIb	n = 6	n = 20
		Hyperflexion	IV	IV	n = 2	n = 6
	Hyperextension	Normal	III	III	n = 6	-
		Hyperextension			n = 1	-
		Hyperflexion			n = 2	n = 2
Normal Pattern *					n = 41	-

MC, metacarpal; MCP, metacarpophalangeal joint; IP, interphalangeal joint; House thumb, House classification of thumb-in-palm; * Normal pattern indicates that thumb-in-palm pattern was only observed when grasping a small object.

**Table 3:** Regression analysis of SHUEE subscales associated with MUUL.

	MUUL Dexterity				MUUL Total			
Variable	Univariable Analysis		Multivariable Analysis		Univariable Analysis		Multivariable Analysis	
	ß (SE)	*p*-Value	ß (SE)	*p*-Value	ß (SE)	*p*-Value	ß (SE)	*p*-Value
**SHUEE**								
DPA (thumb, %)	0.15 (0.02)	<0.001 *	0.05 (0.03)	0.085	0.57 (0.06)	<0.001 *	0.19 (0.10)	0.067
DPA (finger, %)	0.09 (0.02)	<0.001 *	−0.01 (0.02)	0.685	0.32 (0.06)	<0.001 *	−0.06 (0.06)	0.307
SFA (%)	0.26 (0.03)	<0.001 *	0.15 (0.05)	0.002 *	0.97 (0.09)	<0.001 *	0.59 (0.15)	<0.001 *
GRA (%)	0.21 (0.03)	<0.001 *	0.07 (0.03)	0.033 *	0.77 (0.09)	<0.001 *	0.28 (0.11)	0.011 *

SE, standard error; MUUL, Melbourne Assessment of Unilateral Upper Limb Function; SHUEE, Shriners Hospital Upper Extremity Evaluation; DPA, dynamic positional analysis; SFA, spontaneous functional analysis; GRA, grasp release analysis; * *p* < 0.05 by linear regression analysis.

## Data Availability

Data available on request due to privacy/ethical restrictions.
